# Differentials and predictors of food insecurity among Federally Qualified Health Center target populations in Philadelphia: a cross-sectional study

**DOI:** 10.1186/s12889-023-16208-3

**Published:** 2023-07-10

**Authors:** Kai Inguito, Brandon Joa, James Gardner, Eric N. Fung, Laura Layer, Karen Fritz

**Affiliations:** 1 Sidney Kimmel Medical College, Thomas Jefferson University, Philadelphia, PA USA; 2 Department of Theology and Religious Studies, Villanova University, Villanova, PA USA; 3 Department of Family Medicine, Naval Hospital Camp Pendleton, Oceanside, CA USA; 4 Philadelphia College of Osteopathic Medicine, Philadelphia, Pennsylvania USA; 5 Department of Epidemiology, Harvard T.H. Chan School of Public Health, Boston, MA USA; 6 Esperanza Health Center, Philadelphia, PA USA; 7 Former: Department of Pediatrics, Drexel University College of Medicine, Philadelphia, PA USA

**Keywords:** Food insecurity, Underserved populations, Social determinants, Geographic health disparities

## Abstract

**Background:**

Over the past decade, the prevalence of food insecurity declined in the United States but curiously climbed in Philadelphia, Pennsylvania, a sizable metropolitan area where many households experience food insecurity and are dependent on programs like SNAP. Therefore, we aimed to determine the burden of food insecurity among populations near Philadelphia Federally Qualified Health Center (FQHC) clinic sites.

**Methods:**

This cross-sectional study was conducted in North Philadelphia, a populous and impoverished section of Philadelphia with many zip codes reporting 30–45% or more of the population below the federal poverty line. Students and clinicians affiliated with a local FQHC conducted surveys on residents (*n* = 379) within 1-mile radiuses of three FQHC sites, using the Hunger Vital Sign™, a validated food security tool. Survey data were collected through door-to-door visits in the summer of 2019. We used simple, age-adjusted bivariable, and multivariable logistic regression models to predict food insecurity with independent variables, including age, sex, language preference, and BMI category.

**Results:**

Food insecurity in North Philadelphia was much higher (36.9%) than previously reported in Philadelphia and nationwide. Food insecurity was inversely associated with age (AOR = 0.98, 95% CI: 0.97, 1.00), overweight (AOR = 0.58, 95% CI: 0.32, 1.06), and obesity (AOR = 0.60, 95% CI: 0.33, 1.09).

**Conclusion:**

In North Philadelphia, the burden of food insecurity is higher than in the greater Philadelphia area, Pennsylvania state, and the rest of the nation and is predicted by age and BMI of residents. These findings demonstrate a need for more locally targeted research and interventions on food insecurity in impoverished urban settings.

## Background

Food insecurity is a condition in which people lack access to food due to insufficient money, geographical location, or other factors. It is a pressing problem especially in urban areas alongside obesity and malnutrition, affecting around 50 million Americans [1, 2]. Lack of access to healthy food options contributes to food insecurity for people in lower income urban areas, where calorie-dense and nutrient-poor options are more plentiful and convenient [3]. Food insecurity is associated with various chronic conditions including cardiovascular disease, developmental delays, asthma, depressed immune function and increased hospitalizations, etc., which FQHC primary care practitioners must address and prevent [4–6]. FQHCs have previously been helpful with gathering public health data [4] through closer contact with surrounding patient populations and continuity of care for patients’ specific, localized needs. In the United States, being black or Hispanic, being female, and being obese are factors associated with greater risk for food insecurity; the complex interplay between behavioral and physiological mechanisms relating obesity and food insecurity is still under investigation [7, 8]. Typical national profiles of food-insecure persons do not consider more specific regional differences, such as at the city and zip code level [7].

Compared to the nation, Philadelphia is a major metropolitan area with higher rates of poverty, food insecurity (18.6% vs 11.8 nationally) [6, 9], and diseases related to food insecurity [6, 10]. In Philadelphia, people of Hispanic background, female, aged 40–59, and enrolled in the Supplemental Nutrition Assistance Program (SNAP, previously called food stamps) are more likely to be food insecure [11]. North Philadelphia is a particularly impoverished area of Philadelphia known as an epicenter of the opioid epidemic [12], with many transient visitors arriving in the area for opioid use and exchange. In a given area, associations of food insecurity with conditions like diabetes or substance use disorders can make targeting co-occurring conditions an effective strategy for local programs’ resource use. Food insecurity prevalence has been estimated as high as 30% throughout North Philadelphia’s fifteen zip codes overall [10]. Federal support programs such as SNAP and WIC (Special Supplemental Nutrition Program for Women, Infants, and Children), and local initiatives [13–15] address healthy food affordability, geographic disparities such as food deserts, and co-occurring diseases through interventions such as subsidies and tailored diets [1, 11, 16]. Yet even with government interventions, food security from 2012–2017 increased by over 20% in Philadelphia while it declined on a national level [17–19].

To inform organizations’ programming on food insecurity and contribute to a more accurate understanding of how food insecurity manifests in a low-income district of Philadelphia, we conducted a cross-sectional study of individuals in North Philadelphia. We hypothesized that, in keeping with findings on a national level, people who were older, female, spoke Spanish, and overweight or obese would be more likely to be food insecure.

## Methods

### Study design and setting

This study was designed as a cross-sectional study examining food insecurity prevalence and its relationship with age, sex, language(s) spoken, and BMI. The study was conducted in three zip codes of North Philadelphia during the period spanning from June–August 2019.

The present study included North Philadelphia residents, aged 18 years and older, who live within a one-mile radius of the sites of EHC. We administered food insecurity surveys to randomly selected individuals primarily in the three zip codes surrounding the EHC sites—19,133 West Kensington, 19,134 Kensington, and 19,140 Hunting Park. Blocks in these zip codes were chosen at random with each house on selected blocks given opportunity to participate. Children screened through the FQHC outreach were not included in the study. Using Cochran’s formula, a sample size of 345 individuals was calculated for a 95% confidence interval. Data were de-identified prior to statistical analysis and reporting, and therefore the Thomas Jefferson University Institutional Review Board provided the study with exempt status.

### Main outcome measure

The main outcome of interest was food insecurity, which is defined as not having reliable access to food. Screening tools derived from the U.S. Household Food Security Survey Module, promulgated by the USDA, have good sensitivity and specificity for detecting food insecurity and measuring severity [20]. We therefore considered validated USDA-derived tools to assess our study population in the context of the aforementioned food insecurity figures and their studies, which also used these tools. The Hunger Vital Sign™ (HVS) is a validated 2-question screening tool that does not impose a high response burden and is suitable for measuring and characterizing prevalence when measuring severity is not a priority [21]. The tool has been determined to have a sensitivity of > 95% and a specificity of 93% for the general national population, with a specificity of > 74% to > 86% for special groups depending on the target population’s age, their income level, and whether they had children [22, 23]. The surveyed neighborhoods included but were not limited to individuals over 60 (previously projected specificity 89%), individuals under the federal poverty line (specificity 80%), and people with children (specificity 80%), although we did not collect data on income level or children for the purposes of this study [23].

Food secure (FS) was designated negative and food insecure (FI) was designated positive. The Hunger Vital Sign™ screening tool asks people whether the following statements are “never true,” “sometimes true,” or “often true”: Question 1, “Within the past 12 months we worried whether our food would run out before we got money to buy more,” and, Question 2, “Within the past 12 months the food we bought just didn’t last and we didn’t have money to get more”. Answering one or both of these statements with “sometimes” or “often” indicates a positive screen.

### Independent variables

Independent variables included BMI (kg/m^2^) and the self-reported sociodemographic factors of age, sex, primary language, and zip code. BMI was categorized according to the standard World Health Organization groupings of underweight (< 18.5), normal (18.5–24.99), overweight (25–29.99), and obese (> 30) [24]. Those who were bilingual English and Spanish speakers who indicated no preference for Spanish (i.e., not exclusively Spanish-speaking) were considered English speakers.

### Data collection

Teams of trained health professional students who participated in an EHC-sponsored program called The Summer Medical Institute (SMI) of Medical Campus Outreach Philadelphia performed door-to-door health screenings in North Philadelphia in the summer of 2019. Upon reaching each selected block, each house was contacted through door knocking until contact had been attempted for all houses on the block. Individuals on the streets and parks and homeless individuals were included in the screenings. One questionnaire was completed per household or per individual for those on streets. Since the neighborhood health screenings occur every summer, food insecurity surveys were implemented in the summer of 2019 as part of the protocol. We included the Hunger Vital Sign™ screening tool as well as height and weight measurements. To account for respondent schedules, surveys were also completed over the weekend, and residences were revisited if there had been no answer on a previous attempt. Interpreters were used to transmit information to participants who exclusively spoke Spanish.

After obtaining written consent, demographic information of survey respondents was collected. To determine BMI, weight (kg) was measured via portable scales and height (m) was obtained via measuring tape. If measurements were unable to be performed, self-reported weight and height were recorded. In accordance with the mission of EHC and FQHCs generally, our study methodology was designed to harmonize with assessment of other social determinants of health and the establishment of continuity of care.

Researchers attempted to contact 2488 houses and passersby for screening and surveys, and 1611 individuals of the total houses knocked did not answer. Of the 877 responders who interacted, 498 declined to participate in the food insecurity survey and 379 completed the survey. These response rates were consistent with those for health screenings in previous years and those of similar published studies (see [25]). Individuals who screened positive for food insecurity were given a list of local food resources and were offered a referral to SNAP, and all people screened regardless of food security status were referred to Esperanza Health Center to establish continuity of care if they did not already have a primary care practitioner.

### Statistical analysis

First, we tabulated summary statistics of the food insecure and food secure groups. For age and continuous BMI, we calculated mean values with standard deviation (SD) for both groups, as well. We also conducted two-sample *t*-tests to determine whether characteristic differences between the food insecure and food secure groups were statistically significant.

Second, we used unadjusted simple logistic regression analysis to calculate crude odds ratios for the specific associations between the dependent dichotomous variable food insecurity and age, sex, language spoken, and weight (BMI) category, individually. That is, each independent variable was individually regressed upon food insecurity. For all analyses that adjusted for weight category, we designated “normal weight” (BMI = 18.5–24.9) as the reference group. The basic logistic regression equation takes the form:

$$\mathrm{ln}(\frac{p}{1-p})={\upbeta }_{0}+{\upbeta }_{1}{x}_{1}$$

Here, the argument (*p* / 1 – *p*) of the natural logarithm function is the odds of food insecurity. The constant β_0_ is the log odds of food insecurity when *x*_*1*_ = 0. The independent variable *x*_*1*_ represents either age, sex (male = 1), language (English = 1), or BMI category. All of the unadjusted odds ratios were obtained by exponentiating β_1_. To obtain a more interpretable odds ratio for the association between age and food insecurity, we exponentiated the odds ratio associated with age to the tenth power, thus yielding the odds ratio of food insecurity associated with a 10-year higher age.

Third, given that age is a strong predictor of food insecurity [26], we conducted age-adjusted bivariable logistic regression analyses to quantify the association between food insecurity and (1) age and sex, (2) age and language spoken, (3) age and BMI category. In these age-adjusted models, the equation takes the form:

$$\mathrm{ln}(\frac{p}{1-p})={\upbeta }_{0}+{\upbeta }_{age}{x}_{age}+{\upbeta }_{1}{x}_{1}$$

The independent variable *x*_*1*_ represents either sex, language, or BMI category. Exponentiating β_*age*_ yielded the odds ratio of food insecurity associated with each 1-year increase in age, holding the other independent variable constant. Likewise, exponentiating β_1_ yielded the odds ratio of food insecurity associated with either sex (male vs. female), language (English vs. Spanish), or BMI category (reference = normal weight), adjusting for age.

Fourth, we constructed a multivariable logistic regression model to calculate a fully adjusted odds ratio for the association between food insecurity and all covariates: age, sex, language, and weight category. As with the above models, the equation takes the form:

$$\mathrm{ln}(\frac{p}{1-p})={\upbeta }_{0}+{\upbeta }_{age}{x}_{age}+{\upbeta }_{sex}{x}_{sex}+{\upbeta }_{language}{x}_{language}+{\upbeta }_{BMI}{x}_{BMI}$$

To obtain the fully adjusted odds ratios for the associations between food insecurity and one of the dependent variables, holding the others constant, we exponentiated each of the β coefficients. For example, exponentiating the coefficient β_*sex*_ yielded the odds ratio of food insecurity, comparing males to females, holding age, language, and BMI category constant.

To account for missing data on age (*n* = 5), language (*n* = 3), and BMI category (*n* = 42 [*n* = 25 for food secure, *n* = 17 for food insecure]), we conducted complete case analyses, whenever applicable. In the bivariable models, the age- and language-adjusted model had *n* = 8 missing, and the age- and BMI-adjusted model had *n* = 47 missing. In the fully adjusted model, there were *n* = 50 missing. For all analyses, a *p*-value ≤ 0.05 was considered statistically significant. We considered a *p*-value between 0.05 and 0.10 to be borderline significant. We used Stata, Version 17 (Copyright 1985–2021, StatCorp LP) to conduct all mathematical analyses.

## Results

**Fig. 1:**
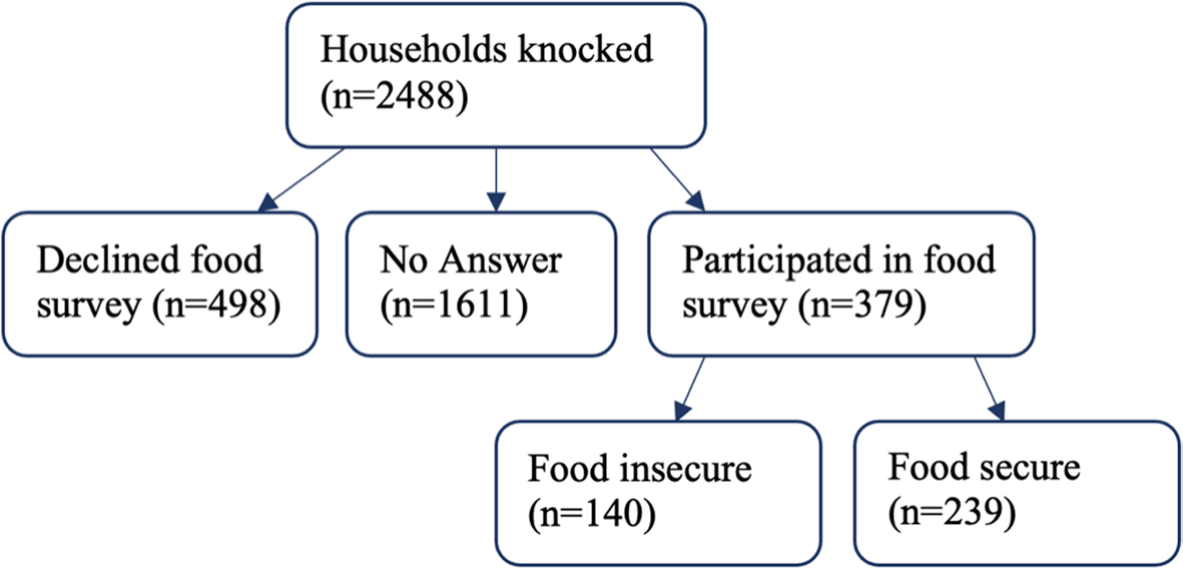
Sample population selection. Out of 2488 attempted contacts, 1611 did not answer, and 877 responded, 498 individuals declined to participate in the food insecurity survey, while 379 individuals agreed to participate. 140 individuals screened positive and 239 screened negative

**Fig. 2:**
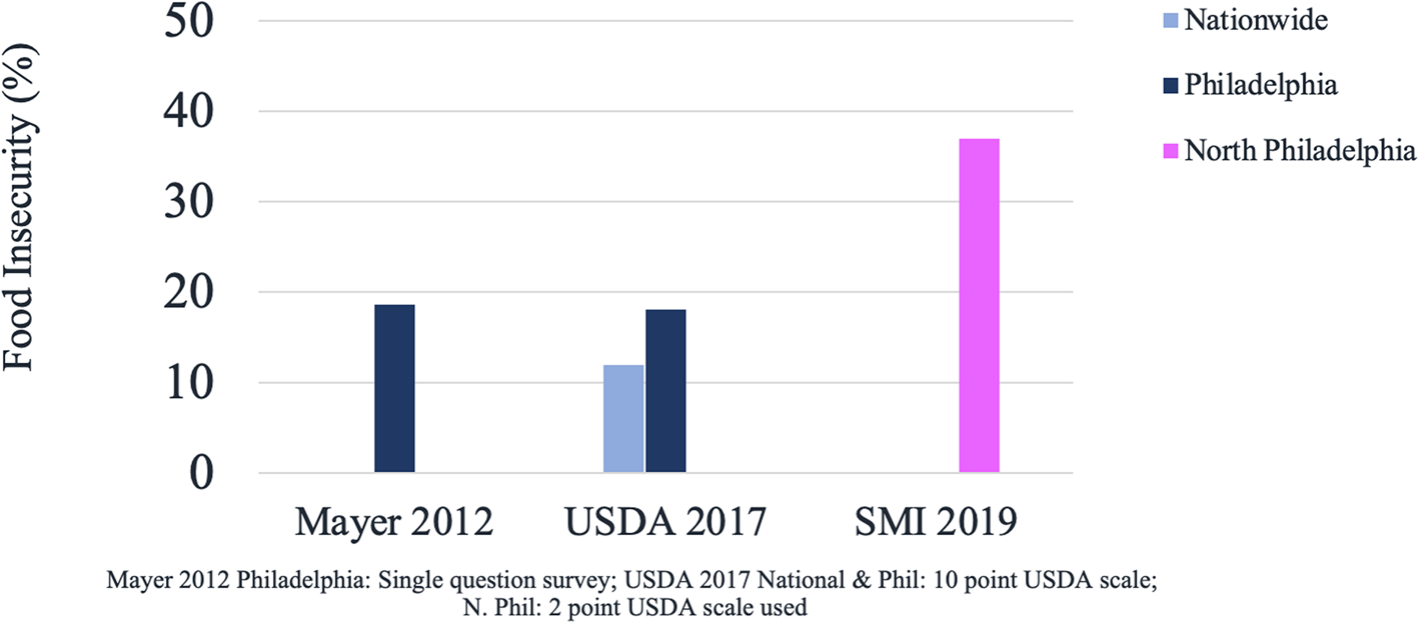
North Philadelphia food insecurity prevalence compared with national and Philadelphia data. **A** Using a one-question screen in 2012, Mayer et al. found FI prevalence of 18.6% in Philadelphia [11]. **B** Using the ten-question USDA survey and in a report by Hunger Free America, FI prevalence in 2017 was 11.8% nationally and 18.3% in Philadelphia [17]. **C** Using the two-question Hunger Vital Sign™ survey, our study found FI prevalence in the screened area in North Philadelphia in 2019 was 36.9%

Of those who completed the survey, 140 screened positive for food insecurity while 239 were negative (Fig. 1, Table 1). Overall, 36.9% of respondents screened positive for food insecurity, compared to 18.3–18.6% in Philadelphia and 11.8% in the general U.S. population (Fig. 2) [11, 12, 17].

**Table 1:** HVS questionnaire responses

			Question 1	
		Never	Sometimes	Often
	Never	*239*	**16**	**3**
Question 2	Sometimes	**7**	**46**	**16**
*Italics* = FS **Bold** = FI	Often	**3**	**7**	**42**

(A) Question 1: “Within the past 12 months we worried whether our food would run out before we got money to buy more.”

(B) Question 2: “Within the past 12 months the food we bought just didn’t last and we didn’t have money to get more

**Fig. 3:**
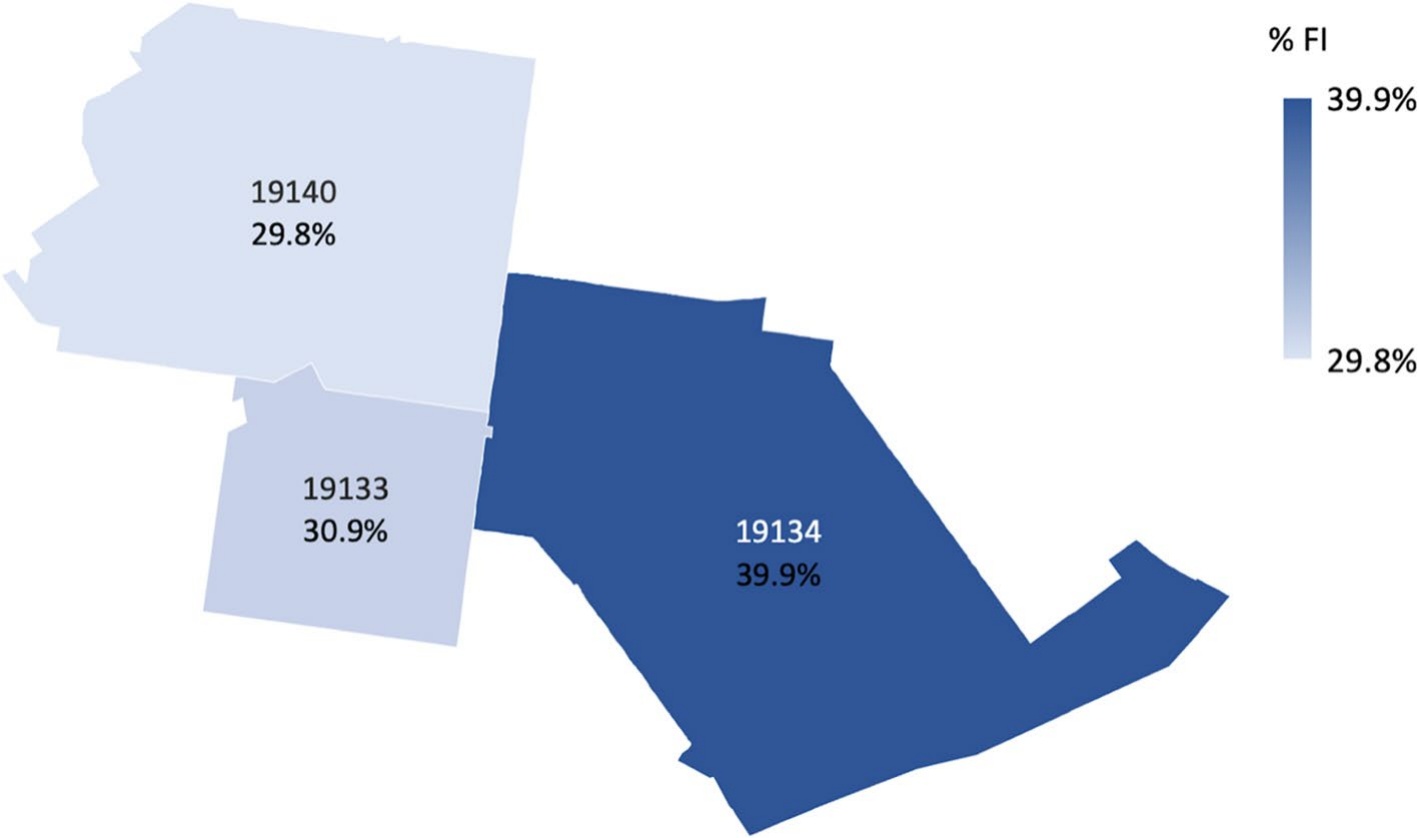
Food insecurity prevalence by zip code. **A** 19133 (*n* = 68) had a FI prevalence of 30.9%, 19,134 (*n* = 158) prevalence of 39.9%, and 19,140 (*n* = 84) prevalence of 29.8%. **B** Out of respondents from all other zip codes (*n* = 63), 46.0% were FI. **C** Map was generated using the Map Chart function in Microsoft Excel 365 (2022)

We grouped survey participants by self-reported zip code and determined the proportion of food insecurity within each zip code, as well as the proportion of all other zip codes combined. Food insecurity proportion ranged from 29.8% to 39.9% by zip code, with an even higher prevalence among those not reporting a local zip code at 46.0% (Fig. 3).

On average, food insecure individuals were younger (47.6 ± 13.5 years) than those who were food secure (52.5 ± 15.1 years) (*p* = 0.002) (Table 2). Those with food insecurity also had lower BMI (27.8 ± 6.0 kg/m^2^) than the food secure individuals (29.5 ± 6.2 kg/m^2^) (*p* = 0.016). There was no difference statistically between the food insecure and the food secure by sex (*p* = 0.212). However, food insecure individuals were more likely to speak English or both (vs. Spanish speakers) (*p* = 0.028).

**Table 2:** Characteristics of food secure and insecure respondents

**Characteristic**	FI (*n* = 140)	FS (*n* = 239)	***P*-value**
***Age (years)***	47.6 ± 13.5	52.5 ± 15.1	0.002
***Sex***			
Male	69 (49.3%)	102 (42.7%)	0.212
Female	71 (50.7%)	137 (57.3%)	
***Language***			
English	82 (59.8%)	115 (48.1%)	0.028
Spanish	55 (40.2%)	124 (51.9%)	
***Weight—Continuous (BMI)***	27.8 ± 6.0	29.5 ± 6.2	0.016
***Weight—Categorical (BMI)***			
Underweight (< 18.5)	3 (2.4%)	1 (0.5%)	0.016
Normal weight (18.5–24.99)	39 (31.7%)	43 (20.1%)	
Overweight (25–29.99)	39 (31.7%)	83 (38.8%)	
Obese (> 30)	42 (34.2%)	87 40.6%)	

(A) Age mean age with standard deviation, *SD* Standard deviation, *FI* Food insecure, *FS* Food secure, *REF* Referent group

Formal simple logistic regression analyses supported the observed differences between the food insecure and food secure groups (Table 3, Models 1–4). The odds ratio for the association between food insecurity and age was 0.98 (95% CI: 0.97, 0.99). In other words, an individual 10 years older had 20% lower odds of food insecurity than another individual. English speakers had 61% (95% CI: 1.05, 2.46) higher odds of food insecurity than Spanish speakers. There was an association between being male and food insecurity, but the result was not statistically significant (COR = 1.31, 95% CI: 0.86, 1.98, *p* = 0.213). Those who were overweight (COR = 0.52, 95% CI: 0.29, 0.92) and obese (COR = 0.53, 95% CI: 0.30, 0.94) had lower odds of food insecurity. Although a large magnitude association was found between underweight and food insecurity (COR = 3.31, 95% CI: 0.33, 33.13), there was insufficient power to reach statistical significance (*p* = 0.309).

**Table 3:** Predictors of food insecurity from simple, age-adjusted bivariable, and fully adjusted logistic regression analysis

**Characteristic**	**Model 1** OR (95% CI) *p*-value	**Model 2** OR (95% CI) *p*-value	**Model 3** OR (95% CI) *p*-value	**Model 4** OR (95% CI) *p*-value	**Model 5** OR (95% CI) *p*-value	**Model 6** OR (95% CI) *p*-value	**Model 7** OR (95% CI) *p*-value	**Model 8** OR (95% CI) *p*-value
Age*	0.98 (0.97, 0.99) 0.002	—	—	—	0.98 (0.96, 0.99) 0.002	0.98 (0.97, 1.00) 0.021	0.98 (0.96, 0.99) 0.008	0.98 (0.97, 1.00) 0.063
Sex** (Male)	—	1.31 (0.86, 1.98) 0.213	—	—	1.30 (0.85, 1.99) 0.216	—	—	1.21 (0.75, 1.93) 0.435
Language** (English)	—	—	1.61 (1.05, 2.46) 0.029	—	—	1.36 (0.87, 2.12) 0.182	—	1.41 (0.86, 2.29) 0.171
Weight***								
Underweight	—	—	—	3.31 (0.33, 33.13) 0.309	—	—	3.08 (0.31, 31.00) 0.341	3.26 (0.32, 33.36) 0.319
Normal	—	—	—	REF	—	—	REF	REF
Overweight	—	—	—	0.52 (0.29, 0.92) 0.025	—	—	0.58 (0.32, 1.06) 0.076	0.58 (0.32, 1.06) 0.079
Obese	—	—	—	0.53 (0.30, 0.94) 0.030	—	—	0.53 (0.30, 0.96) 0.035	0.60 (0.33, 1.09) 0.092

(A) *Models 1–4* simple (unadjusted), *Models 5–7* age-adjusted bivariable, *Model 8* fully adjusted

(B) *OR* Odds ratio. *REF* Referent group

(C) * = continuous, ** = dichotomous, *** = categorical

(D) For Model 1, the 10-year age-associated *OR* = 0.80 (95% CI: 0.69, 0.92)

(E) Chi-square tests: *Model 5*, *p* = 0.004; *Model 6*, *p* = 0.007; *Model 7*, *p* = 0.003; *Model 8, p* = 0.010

In age-adjusted bivariable logistic regression models (Table 3, Models 5–7), the magnitude of the odds ratio for the association between food insecurity and age remained unchanged from the age-only adjusted simple regression—and regardless of whether the second covariate in the model was sex, language spoken, or weight category (AOR = 0.98). After adjusting for age, the association between food insecurity and sex did not change (AOR = 1.30, 95% CI: 0.85, 1.99), and the association between food insecurity and language spoken was attenuated (AOR = 1.36, 95% CI: 0.87, 2.12) and was no longer statistically significant (*p* = 0.182). In the age- and weight-adjusted model (Table 3, Model 7), the associations between food insecurity and obesity (AOR = 0.53, 95% CI: 0.30, 0.96) and overweight (AOR = 0.58, 95% CI: 0.32, 1.06) remained unchanged, though overweight became only borderline significant (*p* = 0.076).

Even after adjusting the model fully for age, sex, language, and weight category in a multivariable regression (Table 3, Model 8), the magnitude of the association between a 1-year age increase and food insecurity was AOR = 0.98 (95% CI: 0.97, 1.00), though the result became only borderline significant (*p* = 0.063). Sex (AOR = 1.21, 95% CI: 0.75, 1.93) and language (AOR = 1.41, 95% CI: 0.86, 2.29) remained statistically not significant. Finally, obesity (AOR = 0.60, 95% CI: 0.33, 1.09) and overweight (AOR = 0.58, 95% CI: 0.32, 1.06) remained inversely associated with food insecurity, but both also became only borderline significant (*p* = 0.092, 0.079, respectively).

## Discussion

In this cross-sectional study of food insecurity among residents of a low-income area of Philadelphia, we found a high prevalence, which exceeded that of other areas in Philadelphia and the country. Food insecurity was found to vary between zip codes. Individual characteristics predicting food insecurity included younger age, English-speaking, and lower BMI, but not a particular sex (Table 3). Younger age was the most consistent and statistically significant contributor to food insecurity out of all variables considered together (Table 3, Model 8). These predictors of food insecurity reflected vulnerabilities among demographics not historically targeted by organizations serving the study area and addressing food insecurity in Philadelphia and the U.S. Our hypothesis that older, female, Spanish-speaking, and overweight and obese individuals would be more likely to be food insecure was thus shown to be incorrect. The findings suggest that some overlooked demographic characteristics should be considered in strategies for addressing food insecurity in similar regions. We attend especially to prevalence, association with younger age, and lower weight.

### Prevalence

Food insecurity prevalence was much higher in the neighborhoods surrounding the clinic sites (36.9%) compared to Philadelphia and the United States, which was expected given the area’s poverty and limited access to food sources (Fig. 2). There was variability in proportions of food insecurity by zip code. Respondents may have had various reasons for remaining in the area without reporting residence in one of the three pictured zip codes, including homelessness and transient visiting (Fig. 3).

At extremes of poverty, measures of a community’s health may differ in many regards compared to what is expected in well-resourced areas. Even if considering a very conservative specificity of 75% for the Hunger Vital Sign compared to the USDA 18-question survey and other screens derived from this, differences in validated screening tools would not account for the higher food insecurity prevalence we report in this area. This is because the percentage differences in food insecurity prevalences between the study area and other surveyed areas far exceeds the possible 25% variability for a 75%-specific tool. With a most conservative estimate, this area has a food insecurity prevalence at least double the national level and over 50% higher than Philadelphia overall. Poverty modifies food insecurity and potentially has a strong effect on the food insecurity proportions seen in this area [27]. We thus speculate that confounders such as high rates of opioid use, extreme poverty, and homelessness contributed to this high proportion of food insecurity, especially among those not reporting a local zip code or who were visiting from outside those three zip codes [28]. These results, on a smaller level, are evocative of much-discussed statistics such as the nearly 20-year life expectancy gap between zip codes within Philadelphia [12]. A previous study had already noted that prevalence of food insecurity in the greater Philadelphia region has sometimes been underestimated due to confounding [11]. Additionally, food insecurity prevalence in the greater Philadelphia area appears to be increasing, as demonstrated in a 2017 study showing 15–18% food insecurity [17, 19].

### Association with younger age

Alongside a higher prevalence, we found different predictors of food insecurity compared with other populations [29]. The most robust predictor was younger age. Language was predictive when considering simple logistic regression models but was shown to be less contributory in bivariable and multivariable analyses (Table 3, Models 3, 6, 8). The study area has a larger proportion of young people and a smaller portion of the population aged 65 years and older (10.5%) [28]. Whereas national data show that food insecure people tend to be older, in this study area, the average food insecure person was 47.6 years old while the average food secure person was 52.5 years old. To reiterate, across the simple, bivariable, and fully adjusted models, each 10 year decrease in age was associated with 20% higher odds of food insecurity for individuals in the study.

Age appears to be a consistent contributor to food insecurity in many studies including this one, motivating its inclusion in the bivariable regression analysis. Further isolating age through the bivariable regression and fully adjusted multiple regression again showed a significant contribution of age to food insecurity, but in the opposite direction to associations found in other populations. Several issues present in the study population could elucidate why older age was protective against food insecurity in this study at the same time that there is a relatively low elderly portion of the population. When speaking to older individuals, most had resided in the area for a longer time and had seen changes brought by successive waves of substance use epidemics. Older individuals may have been more aware of local support programs and organizations promoting food security as their presence in the community had preceded these programs, and some had contributed feedback to their formations. Meanwhile, younger people may be more susceptible to issues such as violence and drug abuse that affect food security and contribute to high mortality and a low life expectancy in the area [28], hence the smaller population of elderly individuals.

Further, the study area exhibits some of the highest rates of opioid abuse and illegal drug selling in Philadelphia [30], the area is known nationally as a hub for drug distribution [12], and these activities were observed on the streets when collecting survey data. Opioid use is more common among English-speaking individuals who are younger [31]. That older age is associated with a preference for Spanish is a potential explanation for why age is a statistically significant predictor of food insecurity in simple, bivariable, and multivariable models, whereas language loses statistical significance in both age-adjusted bivariable and fully adjusted multivariable models. Increasing rates of opioid use disorder, homelessness, and comorbidities [32] among young people could thus be related to the increased incidence of food insecurity among younger members of this population as other studies have demonstrated association between opioid use disorder and food insecurity [33]. Even so, we were unable to screen individuals in the study population for ongoing substance use while conducting the food insecurity survey.

### Weight and other variables

The majority of the food secure and insecure study population was overweight, consistent with local and national trends [7]. However, when comparing underweight, normal weight, overweight, and obese individuals, we can infer from our underpowered underweight category that underweight individuals were more likely to be food insecure in our study (Table 3, Model 4). Underweight and normal weight may have been risk factors for food insecurity because of perceived distance from grocery stores and other sources of food, whether nutritious or not. Typically, obesity is associated with food insecurity either because of consumption of high calorie foods or limited education and other resources [34], but in the study area, there were few establishments where individuals could procure nutritious food, relating in part to violence and drug usage deterring business activity. The association between lower BMI and food insecurity in our study thus reflected more absolute lack of access to food generally rather than inaccessibility only to nutritious food sources.

In the bivariable regression analysis (Table 3, Model 7), age and obese BMI achieved significance at the 0.05 level (i.e., older individuals with higher BMI were less likely to be food insecure). Overweight became borderline significant (*p* = 0.076), but this was partially due to the loss of sample for the complete case analysis. That is, a sample of *n* = 332 for Model 7 was fewer than the 345 participants needed as calculated from Cochran’s formula since some participants did not agree to provide weight. Moreover, although age, overweight, and obese were all borderline significant in the fully adjusted model (Model 8), the point estimates remained relatively unchanged. Again, it is likely that missing data and the choice to conduct a complete case analysis contributed to the borderline significance, given that *n* = 329 for Model 8. Given the smaller than expected sample size for the fully adjusted model, we believe that age, obese, and overweight are still important predictors of food insecurity, even in the fully adjusted model. This finding is likely related to similar conditions discussed when we considered why older age was protective against food insecurity. We speculate that individuals who were obese might have had access to local organizational resources and food sources in a place where these were otherwise scarce. Thus, their relationship with food may have more to do with needing education and developing a lifestyle of healthy eating habits than lacking food absolutely.

Model 7 also supported an association between lower BMI and food insecurity, with underweight individuals at greater risk of being food insecure. Although there was a small sample of 4 underweight individuals, we included those in the underweight category because the presence of underweight individuals precluded a positivity violation. We also did not collapse this underweight population into one BMI < 25 category with the normal weight population to avoid introducing confounding. Because of the small sample size, although it was not surprising that underweight individuals were more likely to be food insecure than not, we cannot make any definitive claims on the current population’s distribution. This study was underpowered for underweight subjects, and future research should include collecting more data on food insecurity and underweight individuals in this population.

Women were not more likely than men to be food insecure, a finding related to several factors such as already existing programs and other social determinants of health. Typically, women are thought to be at greater risk for food insecurity due to several issues including greater likelihood of being severely impoverished and having primary caregiver responsibilities for children [35], but the study area demonstrates social determinants that may raise the food insecurity risks for men to similar levels as those of women. High unemployment (14.5%), violence, and drug usage, contributing to a large life expectancy disparity of over 9 years between men (65.7) and women (74.9), all present a burden preventing men from being food secure [28]. Men affected by food insecurity are also not targeted by programs such as WIC, for example, that are designed to aid women.

Neither language nor sex individually achieved statistical significance in the bivariable or multiple regression models but were included because they are typical predictors of food insecurity. Regarding language, besides the opioid use population being predominantly English-speaking, the health center historically specializes in treating patients from Spanish-speaking backgrounds who live in the surrounding areas. As already discussed, opioid use is more common among English-speaking individuals [31], who will likewise be affected by the factors surrounding drug abuse. With this more locally specific information, local organizations should tailor resources particularly for food security and primary care to other vulnerable populations such as those with histories of drug abuse with more attention to English-speaking males. The wide confidence intervals and larger *p*-values in the more complex models may be an artifact of an underpowered study, resulting from missing BMI data. Even so, the simple logistic regression further demonstrated that in this study area with a large portion of Spanish speakers, individuals who were English-speaking were at a significantly greater risk of food insecurity.

### Strengths and limitations

Strengths of this study include ability to study previously overlooked populations whose characteristics may have been collapsed into larger demographic data; comparison between predictive values for interrelated food security variables; demonstration of partnership potential between nonprofits, medical education, and public health research; and the use of a validated tool to evaluate food insecurity prevalence. Programming for food insecurity from state and federal levels may reach unintended populations and therefore fail to meet food needs most effectively if diverse situations in smaller localities remain unaddressed. The differences in expected results on the more local zip code level demonstrate a need for more precise and specific public health research particularly when addressing social determinants of health. Moreover, the work of students was an integral component for gathering and interpreting data while interfacing with the community to give out resources and connect to social service agencies.

This study may therefore serve as a model and proof of concept for partnership between FQHCs and medical education for collecting, assessing, and implementing results from data on social determinants to contribute to public health knowledge [4, 29]. As in our study design, food insecurity surveys could be administered alongside screenings for associated health conditions, such as hypertension, diabetes, and obesity [5, 8, 36] while potentially establishing continuity of care [37]. This study also represents the most recent possible set of data for this population prior to the COVID-19 pandemic, allowing future comparative studies to address how the pandemic has affected food insecurity and possible mechanisms for changes such as increased opioid use or lower employment.

Regarding limitations, there was a possibility for selection bias and reporting bias to have affected results given the low response rate, and generalizability may be limited to similar low-income urban areas in the Northeastern U.S. Response rates were consistent with those for health screenings in previous years and response rates for some similar published studies [25]. Further, since demographic information was not collected for non-respondents, true prevalence of food insecurity may be higher than recorded due to issues like fear of stigma, with previous studies generally noting self-reported values are lower than expected [11]. Other potentially relevant descriptors such as race, socioeconomic status, and access to transportation were not able to be directly considered in our model. Confounding factors such as employment and substance abuse status may explain some differences in findings between food insecurity in the study population from previous municipal and national studies, potentially contributing to food insecurity through less-considered mechanisms. Future studies could develop tactful ways to screen for drug abuse in relevant food insecure populations, perhaps incorporating data from gathering points such as needle exchanges [11, 13]. Clinics and public health agencies should consider addressing food insecurity and confounding barriers simultaneously for greatest effect.

## Conclusion

We conclude that the demographic characteristics and prevalence of food insecurity in this low-income area of North Philadelphia are different from and higher than those on a national level, contributing to an extremely high prevalence of food insecurity in the area. Food insecurity was most consistently inversely associated with higher age and overweight or obesity. Our results support the need for more local and national research on the effects of homelessness and substance abuse on food insecurity prevalence.

## Data Availability

The datasets used and/or analysed during the current study are available from the corresponding author on reasonable request.

## Abbreviations

HVS: Hunger Vital Sign™
FQHC: Federally Qualified Health Center
FI: Food Insecure
FS: Food Secure
SNAP: Supplemental Nutrition Assistance Program
OR: Odds Ratio
COR: Crude Odds Ratio
AOR: Adjusted Odds Ratio
CI: Confidence Interval
BMI: Body Mass Index
